# Physician assistant/associate retirement intent: seeking the exit ramp

**DOI:** 10.1186/s12913-022-08479-0

**Published:** 2022-09-03

**Authors:** Roderick S. Hooker, Andrzej Kozikowski, James F. Cawley, Kasey Puckett

**Affiliations:** 1 Northern Arizona University, Phoenix Biomedical Campus, Phoenix, AZ USA; 2 National Commission On Certification of Physician Assistants, 12000 Findley Road Suite 100, Johns Creek, GA 30097 USA; 3 University of Maryland, Baltimore, MD USA; 4 Florida State University, Tallahassee, FL USA

**Keywords:** Physician assistant, Physician associate, Retirement, NCCPA

## Abstract

**Background:**

Retirement patterns for American physician assistants/associates (PAs) are in flux as the first substantial cadre trained in the 1970s makes their retirement choices. The growing and aging of the US population is increasing the demand for healthcare services. At the same time, provider retirement can decrease patient access to care, disrupt continuity of care and lead to poorer health outcomes. Knowing PA intentions to retire and the retirement patterns can be useful to health system employers and workforce policymakers. The purpose of this study was to investigate the retirement patterns of PAs within the United States. We investigated their characteristics, career roles, and intent to depart from clinical practice.

**Methods:**

Drawing on the National Commission on Certification of Physician Assistants (NCCPA) 2020 health workforce data (*N* = 105,699), the associations of demographics (age, gender, US region, and years certified), and practice attributes (specialty and practice setting) of clinically active PAs were assessed with intending to retire in the next five years. Analyses for this national cross-sectional study included descriptive statistics, Chi-square, and Fisher’s Exact test, as appropriate. A *p*-value of 0.05 or less was considered statistically significant for all analyses where a comparison was made.

**Results:**

Overall, 5.8% of respondents indicated that they intend to retire within five years**.** We detected significant differences (all *p* < 0.001) on intentions to retire by age group, gender, US region, years certified, specialty, and practice setting. Respondents 70 years and older compared to those 60–69 were more likely (66.5% vs. 48.9%), males compared to females (8.8% vs. 4.4%), those who have been certified for more than 21 years compared to 11–20 years (25.6% vs. 4.0%), PAs practicing in family medicine compared to dermatology (7.7% vs. 3.4%) and those in the federal government practice setting compared to rural health clinic (13.6% vs. 9.8%) reported they were more likely to retire in the next five years.

**Conclusions:**

Our study provides a comprehensive snapshot of PA retirement intentions using a robust national dataset. Among the most important factors associated with intent to retire in this study were older age and duration of PA career. Most PAs are remaining clinically active into their seventh decade—suggesting that they are integrated into medical systems that value them and they, in turn, value their role.

## Introduction

The first sizeable group of physician assistants/associates (PAs) who initiated their careers in the 1970s is considering retirement four decades later. Retirement patterns for PAs are in flux and, to date, have not been fully described. PAs often choose to continue clinical practice rather than exiting the clinical workforce when reaching 65 [1]. Before completely retiring, PAs can reduce the number of hours they work or adopt a narrower scope of practice. Such developing patterns have important implications for healthcare delivery as replacement costs can be high [2].

Medical provider retirement patterns are essential to policy planners as shortages loom in the health workforce [3]. Knowing a nation’s healthcare needs and the personnel required to staff health services are critical ingredients of medical labor supply and demand. Predicting the departure of medical personnel from clinical practice permits health systems to develop replacement strategies. In turn, replacement estimates are contingent on intentions to retire and actual exodus rates.

## Background

PAs represent a critical component of the US medical care workforce [4]. As of 2020, there were approximately 149,000 Certified PAs [5]. The number of graduates in 2020 was 11,000 [6]. While the PA pipeline is growing and the career selection of graduates is better understood, the estimates of PA departure patterns are less well known.

One of the first examinations of PA retirement patterns was a 2011 survey (*N* = 625) that found that the average retirement age was 61 years and ranged from 47 to 75 [7]. In that study, PA career duration was 29 years on average, and most PAs were in primary care practices. Most retired PAs indicated they received Social Security, Medicare, and pension. Less than a fifth noted health reasons being a contributing factor for their retirement. Approximately 20% said that they had retired too early and would have preferred to postpone retirement [7]. In examining PAs in the older age bracket (> 65 years), many were working clinically, but to what extent was only superficially discerned [1, 8].

### Physician retirement patterns

In the search for models of retirement patterns, it is helpful to assume that PAs often emulate physician behavior in multiple ways (e.g., specialty choice, attitudes regarding work, lifestyle choices). Activity surrounding pre-retirement and retirement requires multi-faceted decision-making for physicians. It is possible that PAs, like physicians, feel that retirement can be a difficult transition since, in both instances, their personal identity is closely associated with their professions.

The average physician retirement age is typically older than the general population, and retirement considerations often relate to job satisfaction [9]. Other factors may include financial obligations along with the flexibility and autonomy to adjust workload. Also, physicians, particularly those who practice primary care, tend to own/control their practice circumstances to a greater degree than hospital-based physicians [10]. One recent report noted that key physician determinants for retirement include lifestyle preferences, finances, burnout levels, and dissatisfaction with how medicine is practiced today. Over (44%) of physicians indicated lifestyle was the most important factor influencing their choice to retire. The second most frequently reported reason (23% of physicians) was related to finances. Within the survey’s open-ended comments, approximately 20% mentioned being burned out and frustrated with excessive paperwork and patient volume, along with decreased time spent on direct patient care [10].

One large cohort study using administrative data explored pre-retirement activity levels and retirement of Canadian physicians [11]. Nearly 40% retired during the 5-year study period with slow or rapid reductions in practice activity patterns. In the years preceding retirement, almost 40% of physicians decreased their practice activity by 10 to 90% [11].

### Intent to retire

‘Intent-to-retire’ is a term used by human resource administrators and researchers in efforts to predict retirement occurrence [12]. When assessing the retirement of health professionals, intent to retire has significant predictive value. In studies exploring physician retirement, the gap between intention to retire and actual retirement occurrence decreases with increasing age [13, 14].

Understanding the career arc of a PA is essential for workforce planning [15]. While PAs have been a presence in American medicine for half a century, their departure from clinical activity (i.e., patient care as medical providers) is less known. From the entrée into the medical workforce and throughout their career, the employment arc of a PA is becoming more apparent; however, a required piece of information needed is when they will make their employment exit. To this end, we undertook an analysis of the intention to retire for PAs employed in the US.

Research Question: *What is the retirement pattern of American physician assistants/associates?*

## Methods

We utilized data collected by the National Commission on Certification of Physician Assistants (NCCPA). NCCPA provides a mechanism for researchers to request PA workforce data, and only requests that intend to utilize the data for ethical research purposes are approved [16]. NCCPA follows quality assurance protocols to ensure data quality and data provided is de-identified and aggregated to ensure PA anonymity. The data contained administrative and self-reported demographic and clinical practice characteristics of Certified PAs in the US [5]. NCCPA uses an online data gathering instrument, the *PA Professional Profile*, consisting of three modules with optional questions that assess PA demographics and attributes of clinical practice. The *PA Professional Profile,* a comprehensive survey-based instrument*,* was initially launched in 2012. Its development relied on the federal government’s Health Resource and Services Administration’s (HRSA’s) Center for Workforce Studies’ minimum data set (MDS) guidelines. The MDS outlines a framework for gathering essential health workforce questions [17]. The optional algorithm-driven questions are presented to PAs through a secure online portal on the NCCPA website. PAs are encouraged to update the *PA Professional Profile* when they log on to the portal or when entering Continuing Medical Education activity [18].

This study had IRB approval (Sterling IRB# 8759) and focused on a subset of responses collected within the *PA Professional Profile*. The health workforce dataset contained in the *PA Professional Profile* can be retrieved efficiently for various research needs. Specific variables from the *PA Professional Profile* were imported into SPSS via SPSS database queries*.* Of the total number of Certified PAs at the end of 2020 (*n* = 148,560), those who did not update their information in the last three years were excluded. These criteria resulted in 127,560 PAs working across a wide variety of medical and surgical specialties. Of these, 105,699 PAs provided a response to the question assessing intentions to retire in the next five years (83%). The *PA Professional Profile* question added at the beginning of 2018 and used as the primary outcome variable in the study is: *Do you plan to retire from the PA workforce in the next five years?*

The demographic and practice characteristics of the respondents that were assessed on intention to retire in five years included: age, gender, US region, years certified, specialty (total of 70 [26 specialties, 43 subspecialties and other]), and practice setting. For analysis, the specialty variable was collapsed into the most frequently selected nine specialties with the total of all remaining specialties categorized as “other”. Descriptive statistics (counts and percentages) were calculated for all variables. As appropriate, Chi-square tests or Fisher’s Exact tests were conducted on all demographic and practice characteristics to examine if intentions to retire in the next five years were associated with a particular profile. Missing data were excluded from these tests. A *p* value of 0.05 or less was considered statistically significant for all analyses where a comparison was made. Data analyses were conducted using SPSS version 26 (IBM).

## Results

**Fig. 1:**
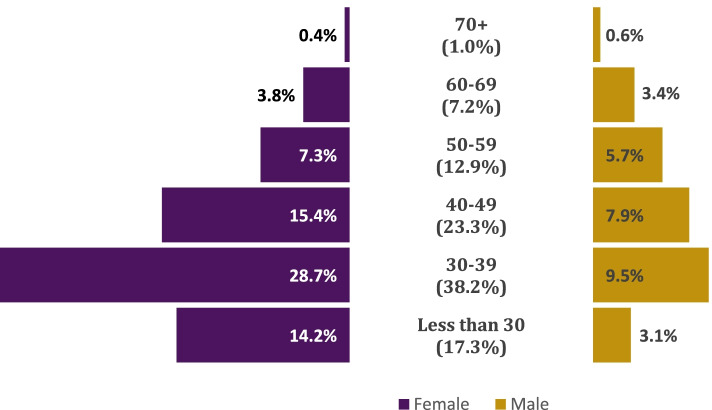
Population pyramid of US PAs 2020

Figure 1 depicts a population pyramid of PA age groups by gender. There were a higher proportion of females in each age group category, except those who were 70 years of age and older. The highest percentage of female and male PAs were in the 30–39 age group, followed by 40–49.

Table 1 summarizes PA demographics, practice characteristics, and intentions to retire. Most PAs were female (69.7%), and over half (56.2%) were certified for up to 10 years. Among all, a majority were 30–39 (38.2%) and 40–49 (23.3%) age groups.

**Table 1:** Demographics, practice characteristics, and intentions to retire of certified PAs

	Percent
Age:	
Less than 30	17.3%
30–39	38.2%
40–49	23.3%
50–59	12.9%
60–69	7.2%
70 +	1.0%
Gender:	
Female	69.7%
Male	30.3%
US Region:	
Midwest	19.7%
Northeast	25.1%
South	34.4%
West	20.9%
Years Certified:	
Up to 10	56.2%
11–20	28.1%
21 +	15.7%
Specialty:	
Surgery – Subspecialties	18.7%
Family Medicine/General Practice	18.1%
Emergency Medicine	12.4%
Internal Medicine—Subspecialties	9.5%
Internal Medicine – General Practice	4.4%
Dermatology	4.1%
Hospital Medicine	3.6%
Surgery – General	3.0%
Pediatric – General	1.9%
All Other Specialties	24.4%
Practice Setting:	
Hospital	41.5%
Office-Based Private Practice	37.7%
Federal Government	5.1%
Urgent Care	5.1%
Community Health Clinic	2.9%
Rural Health Clinic	1.7%
Other	6.0%
Plan to Retire from PA Workforce in the Next Five Years:	
No	94.2%
Yes	5.8%

One-third (34.4%) of Certified PAs resided in the South US region. The top three reported specialties, not including the “other” category of combined specialties (24.4%), were: surgical subspecialties (18.7%), family medicine/general practice (18.1%), and emergency medicine (12.4%). The most common practice setting was a hospital (41.5%), followed by office-based private practice (37.7%). By the end of 2020, 5.8% of Certified PAs indicated that they plan to retire from the PA workforce in the next five years.

Table 2 presents the demographic characteristics and years certified by intention to retire from the PA workforce in the next five years. We found significant differences by age group (*p* < 0.001), gender (*p* < 0.001), and years certified (*p* < 0.001). Respondents aged 70 years and older compared to those 60–69 (66.5% vs. 48.9%), females compared to males (8.8% vs. 4.4%), and those who have been certified for more than 21 years compared to those certified 11–20 years (25.6% vs. 4.0%) reported they were more likely to retire in the next five years.

**Table 2:** Intention to retire from PA workforce in five years by demographic characteristics

	No	Yes	*P*-value
Age:			
Less than 30	99.9%	0.1%	< 0.001
30–39	99.4%	0.6%	
40–49	98.7%	1.3%	
50–59	93.1%	6.9%	
60–69	51.1%	48.9%	
70 +	33.5%	66.5%	
Gender:			
Female	95.6%	4.4%	< 0.001
Male	91.2%	8.8%	
Years Certified:			
Up to 10	99.4%	0.6%	< 0.001
11–20	96.0%	4.0%	
21 +	74.4%	25.6%	

**Fig. 2:**
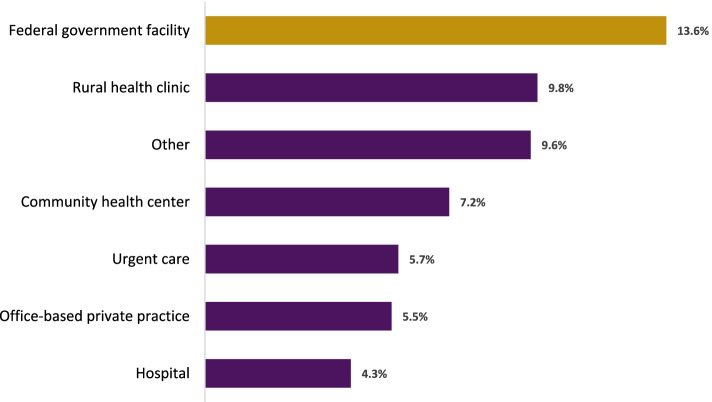
Practice Setting by Intention to Retire in Five Years

PAs employed in the federal government compared to those in rural health clinic practice settings (13.6% vs. 9.8%; *p* < 0.001) were more likely to report they intend to retire in the next five years (Fig. 2). PAs practicing in the hospital setting had the lowest proportion planning to retire in the next five years.

**Fig. 3:**
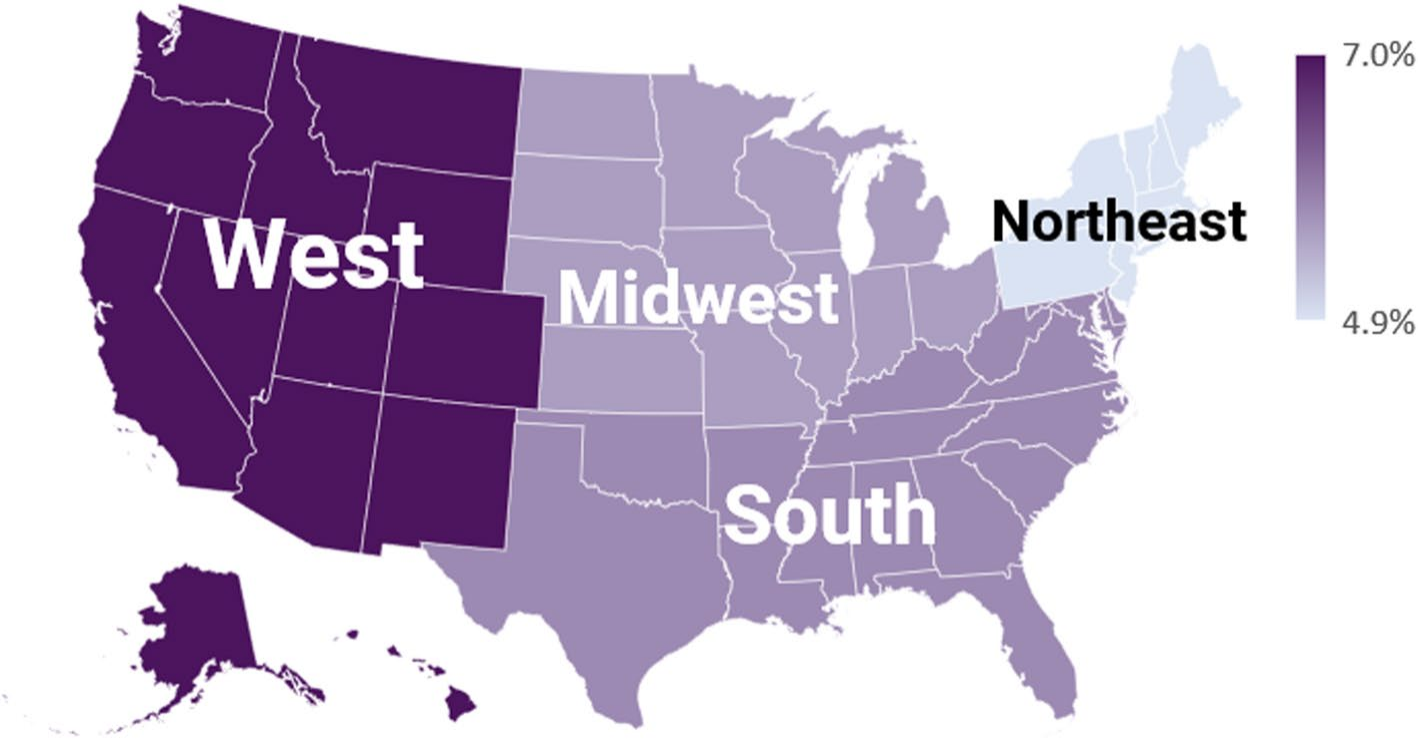
Intention to Retire in Five Years by US Region

We detected statistically significant differences on retirement intention by US regions (*p* < 0.001). PAs in the West US region (7.0%) had the highest proportion of intending to retire, followed by South (5.8%), Midwest (5.6%), and Northeast (4.9%) (Fig. 3).

**Fig. 4:**
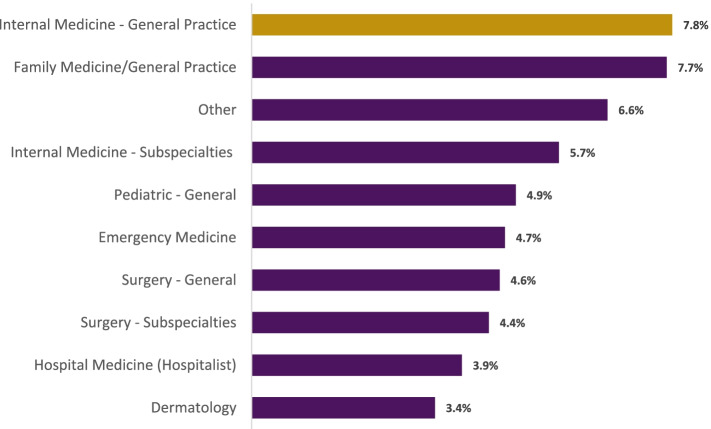
Intention to Retire in Five Years by Specialty

There were also statistically significant differences between PAs practicing in different specialties on their intention to retire (*p* < 0.001; Fig. 4). The top three specialties of PAs that plan on retiring in the next five years included: internal medicine – general practice (7.8%), family medicine/general practice (7.7%), and the total of all other specialties (6.6%).

## Discussion

This investigation set out to identify the demographics and job characteristics of Certified PAs residing in the US intending to retire from clinical activity and to describe newly emerging PA retirement patterns. Knowing the intentions to retire and the age of retirement of PAs represents a new and significant contribution to the body of knowledge related to medical career employment patterns. Such information has ramifications for medical workforce planning and estimates of clinical career output.

In addition, departing the health workforce at any age has been missing in PA studies. Overlooked is that many older providers decide to continue practicing medicine, though not at the equivalent level of activity when fully employed. In fact, retirement for some workers may be part of a range of changes in their career as the individual becomes older. A reduction in workload and narrowing scope of practice may also occur, which have implications for clinician availability. We speculate that PA retirement patterns could resemble those of physicians in that the decision to retire is not a complete cessation of work-related activities. It is probable that some unknown proportion retains some level of clinical practice and related work activities such as teaching, consulting, or other forms of professional work. To what degree and why are questions to be explored.

The PA profession is younger on average than other health professions. PAs have a median age of 38 [5]. According to the Federation of State Medical Boards (FSMB), the average age of all licensed physicians in the US is 52 [19]. Nurse Practitioners (NPs) are 49 years of age on average, and 9.5% are 65 and older [20]. The older an occupation becomes; the more replacement workers are needed.

A systematic review based on 65 studies revealed that physicians retire between the ages of 60 and 69; however, many delay due to financial obligations [14]. A study exploring NPs’ intentions to retire found that the majority (59%) of 60 years and older reported intending to retire in the next five years [21]. In our study, we found that among PAs 60 and older, 51% indicated intending to retire in the next five years.

### Limitations and strengths

The strength of this work is as a new contribution to understanding the PA career [22]. Knowing that most clinically active PAs intend to remain working suggests they are integrated into medical systems that value them and, in turn, value their role. Such new retirement information serves as a keystone integer to predictive modeling and forecasting the availability of the PA in American society.

This study has also had limitations. First, we conducted descriptive and bivariate analyses. We did not conduct multivariate analyses to determine independent predictors of intending to retire in the next five years and their magnitude while controlling for all other covariates such as age, specialty, gender, and years practicing. Moreover, our data did not include reasons for departing early in a career, such as illness, burnout, higher education, administrative roles, or death rate, although we assume it is consistent with pre-COVID-19 national rates. It also did not investigate factors shown to be associated with delayed retirement among physicians, such as job satisfaction and institutional flexibility [23]. The question in the PA Professional Profile asks only if the respondent intends to retire in the next five years. Knowing the full-time equivalent work week leading up to retirement would improve predictive modeling of PAs [24]. This and other intrinsic factors such as professional identity and extrinsic factors for retention or departure need further exploration, especially during a pandemic. As a result, the stage is set to re-examine why some septuagenarian PAs continue working clinically, as well as their gender, urbanity, and specialty [1]. A goal for future research is to utilize multivariate analyses to explore independent predictors of retirement intentions and develop a more granular examination of the complex aspects of PA retirement patterns that include graduated timetables of withdrawal from clinical work, the phenomena of call-back after full retirement, and the profile of post-retirement non-clinical professional activities.

## Conclusion

Intent to retire is a critical piece of labor information needed to understand the career path of the American PA. The characteristics of those remaining or departing facilitate retention strategies for policymakers and human resource managers. Drawing on NCCPA data, it appears that most PAs are remaining clinically active into their seventh decade. At age 60, the predictive value of clinical departure increases among respondents. It also increases for males more than females, and by role: family medicine, general internal medicine, and internal medicine subspecialties. Almost one-sixth of those employed in federal facilities are likely to depart clinical roles, followed by one-tenth in rural health clinics. The research sheds light on PA retirement intentions and sets the stage for future tracking. It also identifies areas needing further investigation, such as institutional flexibility, job satisfaction, burnout and stress management policies in all settings, and identification of predictions for retention and retirement of PAs.

## Data Availability

The datasets generated and analyzed during the current study are not publicly available due to confidentiality of individualized data, but deidentified data can be available if requested from the corresponding author.

## Abbreviations

NCCPA: National Commission on Certification of Physician Assistants
PA: Physician assistant or physician associate
